# Polyprenols of* Ginkgo biloba* Enhance Antibacterial Activity of Five Classes of Antibiotics

**DOI:** 10.1155/2016/4191938

**Published:** 2016-08-23

**Authors:** Ran Tao, Chengzhang Wang, Jianzhong Ye, Hao Zhou, Hongxia Chen

**Affiliations:** ^1^Institute of Chemical Industry of Forest Products, CAF, 16 Suojin Wucun, Nanjing, Jiangsu 210042, China; ^2^Research Institute of Forestry New Technology, CAF, Beijing 100091, China

## Abstract

Polyprenol (GBP) from* Ginkgo biloba* Leaves (GBL) is an important lipid with many bioactive effects. The effect of GBP on antibacterial properties of five antibiotics belonging to different classes was through analysis of inhibition halos, MIC, and FIC index. And we studied the time-killing curves and Ca^2+^ mobilization assay in* Staphylococcus aureus* cells treated with GBP microemulsion and gentamicin sulfate under MIC/2 conditions. These results showed that the GBP microemulsion (average diameter 90.2 nm) combining with gentamicin sulfate had the highest enhancing antibacterial effect against* Staphylococcus aureus*, and the MIC value was 33.0 *μ*g/mL. The increase of the antibacterial effect of tested antibiotics was positively correlated with the decrease of the average diameter of GBP microemulsion. Moreover, GBP microemulsion enhanced antibacterial effect and prolonged antibacterial time of GBP combining with gentamicin sulfate against* Staphylococcus aureus*. GBP microemulsion could enhance the ability of gentamicin inducing an increase in intracellular calcium concentrations to* Staphylococcus aureus*. GBP microemulsion could help some classes of antibiotics to inhibit or kill bacteria. This study supports the fact that GBP microemulsion obviously can not only reduce the dosage of some classes of antibiotics, but also reduce the frequency of the antibiotic use* in vitro*.

## 1. Introduction

*Ginkgo biloba* (Ginkgopsida, Ginkgoales, Ginkgoaceae, and* Ginkgo biloba* L.) is the sole surviving species of the once large plant division Ginkgophyta. Fossils of plants quite similar to* Ginkgo biloba* date back to 180 million years ago and so it has been called a living fossil [1]. Polyprenol (GBP) extracted from* Ginkgo biloba* Leaves (GBL), an important active lipid component, has various medical functions such as antivirus [2], antitumor [3], hepatoprotective [4], and anti-Alzheimer [5] effects and synergistic antibacterial activity [6]. GBP are long-chain polyisoprenoid alcohols. These molecules are typically composed of 15 to 21 unsaturated isoprene units; they are a type of betulaprenol with an* E*,*E*-farnesyl residue at the *ω*-end of the prenyl chain and are terminated by an isoprene unit bearing a primary hydroxyl group [7]. Polyprenyl (in prokaryotes) and dolichyl (in eukaryotes) phosphates act as oligosaccharide carriers during glycan biosynthesis, which is essential to many conserved cellular processes including N-linked protein glycosylation, C- and O-protein mannosylation, and cell wall biosynthesis. Fully unsaturated polyprenols contain all-*trans* isoprene units or a mixture of* trans-* and* cis*-units. Dolichols are distinguished by a single saturated *α*-isoprene unit [8]. The steroid 5*α*-reductase type 3 (SRD5A3), that is, polyprenol reductase, has been found and it could be for the reduction of the alpha-isoprene unit of polyprenols to form dolichols [9]. Bioactivity of polyprenols to the single-celled organisms and the cell of higher organisms* in vitro* gradually became the focus of lipids research. A study determines that alloprenols, a newly discovered class of polyprenols in plants containing an *α*-isoprene unit in the* trans*-(*E*)-configuration, could increase membrane permeability to a greater extent than the more common* cis*-configuration [10]. And some evidence suggests that increased dolichol length can slightly increase membrane destabilization, at earlier time [11]. At earlier study on bioactivity of GBP, we found that GBP with other lipids components including their derivatives could show synergistic antibacterial activity [6]. Based on these studies, it is inferred that maybe GBP was as a development of an effective, a stable, and a safe drug synergist.

In developing countries, overuse of antibiotics cannot be ignored in terms of drug safety. Increasing bacterial resistance to antibiotics and antimicrobials is a growing concern facing the medical, food, and sanitation industries. Formulation scientists are recognizing nanoengineered drug delivery systems as an effective strategy to overcome limitations associated with antibiotic drug therapy. Antibiotics encapsulated into nanodelivery systems will contribute to improved management of patients with various infectious diseases and to overcoming the serious global burden of antibiotic resistance [12]. It is important and meaningful to study the synergistic antibacterial activity of antibiotics combined with GBP. The sesquiterpenoids nerolidol, farnesol, bisabolol, and apritone were investigated for their ability to enhance bacterial permeability and susceptibility to exogenous antimicrobial compounds [13]. The sesquiterpenoid farnesol is one of the important basic materials from biosynthetic pathways of polyprenol and dolichol in plants, animals, and yeasts [14], while nerolidol and bisabolol are isomers of farnesol. Thus we inferred that the study on synergistic antibacterial activity of antibiotics combined with GBP was necessary and possible.

GBP is not soluble in water because it belongs to a hydrophobic lipid with long-chain isopentyl groups. It hinders the biological activity research and related formulation development of GBP. And the study of the biological availability of GBP is of substantial interest, in particular* in vitro* study on the inhibitory effect of some pathogenic microorganisms. Safatov et al.'s study indicated that emulsions of polyprenols that had relatively low hydrophilic-lipophilic balance inhibit influenza virus infection in mice through a modulation of the host immune response [15]. Biopolymer micelles showed significantly improved water solubility/dispersibility and* in vitro* anticancer activity of phytochemicals [16]. This study involves the preparation of oil-in-water type polyprenols microemulsion from GBP with inversed phase emulsification (EIP) method and the effect of GBP on antibacterial properties of five antibiotics belonging to different chemical classes and mechanism of action (ampicillin, ciprofloxacin, gentamicin sulfate, erythromycin, and polymyxin B sulfate). With advance of this study, we would expose the relationship of the effect of GBP on antibacterial properties of five antibiotics with particle size distribution of GBP microemulsion and find out which class of antibiotics had synergistic antibacterial effects combined with GBP. Besides, we study the time-killing curves and the bacteria cell membrane permeability using Ca^2+^ mobilization assay in* Staphylococcus aureus* cells treated with GBP microemulsion and gentamicin sulfate under MIC/2 conditions in this paper.

## 2. Materials and Methods

### 2.1. Materials

**Figure 1:**
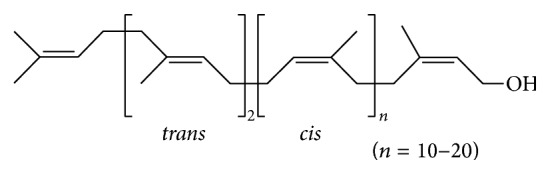
Chemical structures of GBP.

GBP was isolated and purified from GBL in October 2013 from China's Jiangsu Province. GBP (C_70_–C_120_, Figure 1, contents over 98.0%) was determined by HPLC/DAD using an external standard method [6] and the standard polyprenols (C_70_, C_75_–C_105_, C_110_, C_115_, and C_120_) were purchased from Larodan Fine Chemical Co., Ltd. (Solna, Sweden). The five antibiotics were ampicillin (Sigma, A9393, 1 g), ciprofloxacin (Sigma, 17850, 5 g), gentamicin sulfate (Sigma, G3632, 1 g), erythromycin (Sigma, E6376, 5 g), and polymyxin B sulfate (Sigma, P1004, ≥6000 USP units/mg, 5 MU). Emulsifiers were Span 80 (Sigma, 85548, 500 mL) and Tween 80 (Sigma, P1754, 500 mL).* Escherichia coli* (NCTC 12923) and* Staphylococcus aureus* (ATCC 25923) were obtained from the China Center for Type Culture Collection (CCTCC, Wuhan, China).

### 2.2. Extraction, Isolation, and Purification of GBP

GBL (1 kg) air-dried in the shade were pulverized and extracted three times with a total of 3 L of absolute alcohol for 12 h at 75°C; the samples were concentrated to yield extracts (220 g, from GBL) which were then mixed with 1 L 50% NaOH-water solution for 2 h at 85°C. The hydrolysates were extracted three times with 1 L of petroleum ether (b.p. 90~120°C). The solvents were evaporated under vacuum to yield the total nonsaponifiable lipid extracts (42 g, from GBL), which were each dissolved in a solvent mixture (acetone : methanol = 85 : 15, v/v) for a solid : liquid ratio of 1 : 6~1 : 8 (g/mL) and then stored for 2 h at −20°C. The dissolved material was concentrated to yield products in the form of a red oil. The red and brown oils were fractionated by molecular distillation at feed temperature of 60°C, distillation temperature of 280°C, feed flow rate of 180 mL/h, scraper rate of 300 rpm, and operating pressure of 0.1–0.5 Pa to yield heavy distillates in the form of a dark red oil (26 g). GBP was further purified from a portion of the heavy distillates by flash column chromatography (Merck, Kieselgel 60; 0.063~0.2 mm particle size; 4 × 60 cm), using petroleum ether (b.p. 60~90°C) (5 × 150 mL) and 1%, 2%, 3%, and 4% ethyl ether/petroleum ether (5 × 250 mL) as eluents. GBP (1.3 g, 98% by HPLC/DAD) was obtained from the petroleum/ethyl ether (97% : 3%, v/v, GBP) portion [6].

### 2.3. Preparation of GBP Microemulsion

The preparation of oil-in-water type GBP microemulsion was used by inversed phase emulsification (EIP) method [17]. Emulsifiers were Span 80 : Tween 80 (0.514 : 0.486, wt/wt, HLB [hydrophilic-lipophilic balance] value = 9.5) added slowly to 10% GBP solution in n-hexane (emulsifiers : GBP = 1.75 : 1, wt/wt) and stirred with low speed at 40°C using a blender (IKA-WERK, RW 20 DZM, Staufen, Germany) until without n-hexane. After mixing emulsifiers and GBP well, add slowly 8~10 times of water to the mixture until phase inversion. After stirring with low speed at 40°C for 1 h, up to a predetermined temperature, the microemulsion was prepared through stirring with a predetermined high speed for a predetermined time using high-speed agitator (IKA-WERK, Ultra-Turrax, T25 basic, Staufen, Germany). The four variants of GBP microemulsion having different particle size distribution were prepared, that is, GBP-1 (mixing time was 12 min, emulsionizing temperature was 60°C, and mixing speed was 17000 r/min, the same below), GBP-2 (10 min, 60°C, 10000 r/min), GBP-3 (15 min, 75°C, 20000 r/min), and GBP-4 (5 min, 45°C, 10000 r/min), respectively.

### 2.4. Characterization of GBP Microemulsion

In transmission electron microscopy (TEM) analysis, the prepared microemulsion was diluted 10 times with distilled water and placed on a carbon-coated copper grid. Excess phosphotungstic acid was removed with a filter paper. Images were obtained using a TECNAI-10 TEM (Philips, Amsterdam, Holland). For determination of particle size distribution (Mastersizer 2000, Malvern Instruments, Malvern, UK), the samples were prepared that 5 mL prepared GBP microemulsion was diluted with some deionized water. The prepared microemulsion was analyzed by laser particle size analyzer, determined in the range of 0.1~5000 nm at 25°C. For determination of zeta potential (Zetasizer Nano-ZS90, Malvern Instruments, Malvern, UK), the prepared microemulsion was diluted 50 times with distilled water and the samples were measured at a scattering angle of 90° relative to the source using a cascade of photodiode detectors (refraction coefficient was 1.33) at 25°C. Each value reported is the average of three measurements.

### 2.5. Determination of Antibacterial Activity

*Escherichia coli* and* Staphylococcus aureus* were grown in Mueller-Hinton Broth (Sigma, for microbiology) at pH 7.4. Antibacterial tests were carried out using Oxford cup assays [18]. The samples were the four variants of GBP microemulsion (GBP-1, GBP-2, GBP-3, and GBP-4, 500 *μ*g/mL, resp.) with the five antibiotics (10 *μ*g/mL ampicillin, 10 *μ*g/mL ciprofloxacin, 10 *μ*g/mL gentamicin sulfate, 15 *μ*g/mL erythromycin, and 10 *μ*g/mL polymyxin B sulfate, all dissolved in the prepared microemulsion; see the section above) [13] and the resulting solutions were added to Oxford cups which were placed at equal distances on the agar surfaces. The zone of inhibition for each concentration was measured after 24 h incubation at 37°C. The same procedure was repeated in triplicate. Five antibiotics (ampicillin, ciprofloxacin, gentamicin sulfate, erythromycin, and polymyxin B sulfate) were used as positive control and a blank microemulsion (only water with emulsifiers, no GBP, and antibiotics) was used as the negative control. After 24 h of incubation, the diameter of the inhibition halos was observed (measured in mm including cup size). All tests were performed in triplicate and observed values of the inhibition halos are expressed as mean values with standard error of the means (SEM) and analysis of variance (single-factor ANOVA, Tukey's test at 5% probability) [6].

### 2.6. Determination of Minimum Inhibitory Concentration (MIC) and FIC (Fractional Inhibitory Concentration) Index and Determination of the Type of Interactions of Antibacterial Activity

The MICs were determined using the broth-dilution method at ten sample concentrations obtained via serial dilution (256, 128, 64, 32, 16, 8, 4, 2, 1, and 0.5 *μ*g/mL). Solutions showing no visible cell growth were subcultured and incubated at 37°C overnight. The FIC of a factor is the concentration that could be bacterial inhibition when used in combination with another agent divided by the concentration that has the same effect when used alone [19]. The FIC index for the combination of A and B is the sum of their individual FIC values. The determination of the type of interactions referred to synergistic effect (0 < FIC index ≤ 0.5), additive effect (0.5 < FIC index ≤ 1), indifferent effect (1 < FIC index ≤ 4), and antagonism effect (FIC index > 4) [20].

### 2.7. Time-Killing Curves for Gentamicin Sulfate and GBP-1 and Their Mixture against* Staphylococcus aureus* under MIC/2 Conditions

Time-killing curves [21] studies on gentamicin sulfate and GBP and their mixture against* Staphylococcus aureus* under MIC/2 conditions (gentamicin sulfate 2 *μ*g/mL and GBP 64 *μ*g/mL and their mixture containing 0.5 *μ*g gentamicin sulfate and 16 *μ*g GBP per mL) were conducted in duplicate on separate hours. An overnight culture of the isolate was diluted 30-fold with prewarmed Ca-MHB and incubated at 37°C until it reached late-log-phase growth. The bacterial suspension was diluted with Ca-MHB based on the absorbance at 630 nm; the concentration of the bacterial suspension in each flask was approximately 10^9^ CFU/mL. The experiment was conducted for 24 h in a shaker water bath set at 37°C. A sample was taken every 2 h for the first 12 h and every 6 h from 12 to 24 h. Total bacterial populations were quantified by spiral plating 10-fold serial dilutions of the samples (50 *μ*L) onto MHA plates. The plates were incubated in a humidified incubator (37°C) and the bacterial density of each sample was determined using a CASBA-4 colony scanner and software.

### 2.8. Ca^2+^ Mobilization Assay in* Staphylococcus aureus* Cells Treated with Gentamicin Sulfate and GBP-1 and Their Mixture under MIC/2 Conditions

10^6^ CFU/mL* Staphylococcus aureus* cells were centrifuged twice (10 min, 5000 r/min, at 4°C) in PBS and resuspended in 1 mL of PBS. Intracellular calcium was labelled by adding 2 mL Fluo-3-AM to the cell suspension. Cells were incubated for 20 min at room temperature on a shaking table, centrifuged twice, and suspended in PBS. The cell suspension was incubated at 37°C in the dark for 30 min. Every well of the 96-well plates was loaded with 100 *μ*L (total volume) containing the prepared cell suspension and the tested samples under MIC/2 conditions (gentamicin sulfate 2 *μ*g/mL and GBP 64 *μ*g/mL and their mixture containing 0.5 *μ*g gentamicin sulfate and 16 *μ*g GBP per mL). And the fluorescence was collected at 30-second intervals and measured at *λ*_ex_ = 405 nm and *λ*_em_ = 418 nm with a Varioskan Flash multimode plate reader (Thermo Fisher Scientific, Finland). The fluorescence is expressed as mean values with standard error of the means (SEM) and analysis of variance (single-factor ANOVA, Tukey's test at 5% probability) [6].

## 3. Results and Discussion

### 3.1. Preparation of Characterization of GBP Microemulsion

In our previous studies, the preparation process of GBP microemulsion has been optimized using response surface method (RSM) on the study of single factor including HLB value and proportion of emulsifiers, mixing time, emulsionizing temperature, and mixing speed [22]. The result showed that the optimum preparation of polyprenols microemulsion conditions was as follows: emulsifiers were Span 80 : Tween 80 (0.514 : 0.486, wt/wt, HLB value = 9.5), mixing time was 12 min, emulsionizing temperature was 60°C, and mixing speed was 17000 r/min. Besides, the stability and dispersity of polyprenol nanoemulsion under the optimum conditions showed good uniformity at room temperature (more than 90 days) and freeze-thaw (−10°C, more than 80 hours, total) for a certain time in the previous study [22]. Based on the previous study, we selected four sets of conditions (having different mixing time, emulsionizing temperature, and mixing speed with same emulsifiers, HLB value = 9.5) to prepare four variants of GBP microemulsion having different particle size distribution, and it was important to study the relationship of the effect of GBP on antibacterial properties of five antibiotics with particle size distribution of GBP microemulsion.

**Figure 2:**
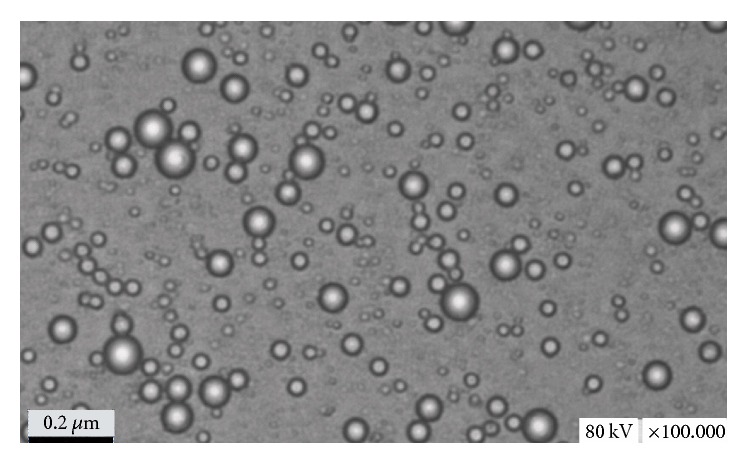
The transmission electron micrograph of GBP-1 microemulsion.

**Figure 3:**
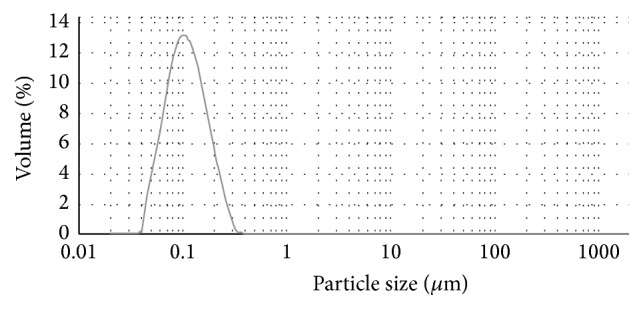
The figure of particle diameter distribution of GBP-1 microemulsion.

The GBP microemulsion was formed when the liquid became clear. The edge of droplets of GBP microemulsion appeared dark, and the centre of droplets appeared bright. A “positive” image was observed using TEM (Figure 2). The results of Table 1 showed that the average diameters and the zeta potential of the four variants of GBP microemulsion (GBP-1, GBP-2, GBP-3, and GBP-4, resp.) were 90.2 nm (Figure 3), −55.2 mv; 289.7 nm, −49.5 mv; 632.9 nm, −45.6 mv; and 11012.2 nm, −29.8 mv (Table 1), respectively. Some active compounds were dispersed in nanoemulsions or polymer micelles-based delivery systems which had shown enhanced oral bioavailability and biological efficacies (anti-inflammation, anticancer, and so on) of different phytochemicals [16]. So these preparations of different variants of GBP microemulsion having different average diameters could help us to reveal the relationship of the effect of GBP on antibacterial properties of five antibiotics with particle size distribution of GBP microemulsion.

### 3.2. Determination of Antibacterial Activity and Synergistic Effects

In this study, the selected five antibiotics belong to different chemical classes and different mechanisms of antibacterial action, respectively. It was useful to reveal the relationship of the effect of GBP on antibacterial properties of five antibiotics with particle size distribution of GBP microemulsion and find out the antibacterial effects of which class of antibiotics could be enhanced. The antibacterial activities of four variants of GBP microemulsion (GBP-1, GBP-2, GBP-3, and GBP-4, 500 *μ*g/mL, resp.) with five antibiotics (10 *μ*g/mL ampicillin, 10 *μ*g/mL ciprofloxacin, 10 *μ*g/mL gentamicin sulfate, 15 *μ*g/mL erythromycin, and 10 *μ*g/mL polymyxin B sulfate) [13] in microemulsion against* Escherichia coli* and* Staphylococcus aureus* were assessed, and their potency was quantitatively assessed based on inhibition halos (Table 2). Analysis of variance (Tukey's test at 5% probability) indicated statistical difference (*p* < 0.05) among all the samples. Among the samples tested, it was indicated that GBP-1 had the highest effect on antibacterial properties of five antibiotics (*p* < 0.05) in addition to combining with ampicillin and polymyxin B sulfate against* Escherichia coli* (combining with erythromycin against* Escherichia coli* and polymyxin B sulfate against* Staphylococcus aureus* was insignificant, the same below). And GBP-2 and GBP-3 showed second and third effect on antibacterial properties of five antibiotics (*p* < 0.05) in addition to combining with ampicillin and polymyxin B sulfate against* Escherichia coli*, respectively. GBP-4 exhibited no effect on antibacterial properties of five antibiotics. It is inferred that the increase of the antibacterial effect of tested antibiotics was positively correlated with the decrease of the average diameter of GBP microemulsion. So GBP-1 (having the smallest average diameter of GBP microemulsion) was selected to study MIC values (Table 3) and FIC index (Table 4) for both bacteria sensitive to five classes of antibiotics with the kind of GBP microemulsion.

The results from Table 4 showed that GBP-1 with gentamicin sulfate exhibited synergistic antibacterial effect (the FIC index of 0.5) compared to GBP-1 with other tested antibiotics (the FIC index range of 0.8~3) against* Staphylococcus aureus*, and the MIC values for* Staphylococcus aureus* sensitive to the GBP-1 with gentamicin sulfate (inhibition halos 25.8 ± 0.1 mm) were 33.0 *μ*g/mL (GBP-1 of 32.0 *μ*g/mL; gentamicin sulfate was 1.0 *μ*g/mL). GBP-1 with ciprofloxacin, gentamicin sulfate, and erythromycin against* Escherichia coli* and with all the tested classes of antibiotics against* Staphylococcus aureus* exhibited additive effect; the FIC index was in the range of 0.8~1. And the GBP-1 with ampicillin and polymyxin B sulfate against* Escherichia coli* exhibited indifferent effect (the FIC index was 3 and the MIC values were up to 130). From the FIC index results, we reached a conclusion that the effect of GBP on antibacterial properties of five antibiotics against Gram-positive bacteria (the FIC index range of 0.5~1) was better than that against Gram-negative bacteria (the FIC index range of 1~3).

### 3.3. Time-Killing Curves for Gentamicin Sulfate and GBP-1 and Their Mixture against* Staphylococcus aureus* under MIC/2 Conditions

**Figure 4:**
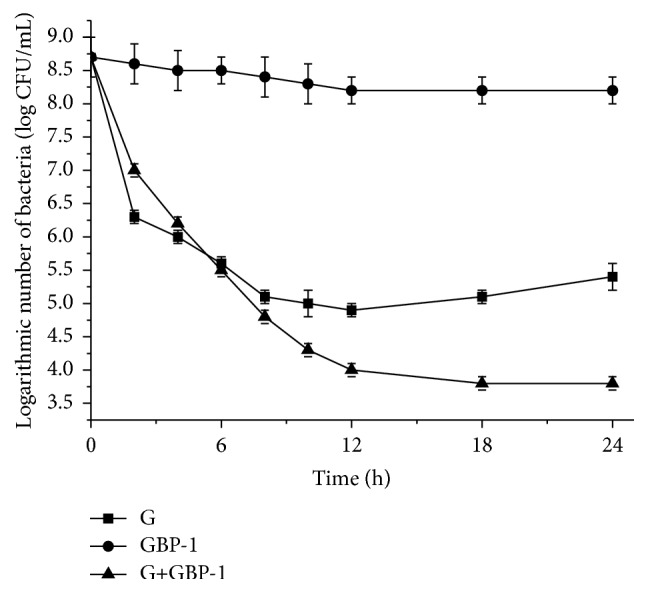
Time-killing curves for gentamicin sulfate (G) and GBP-1 and their mixture against* S. aureus* under MIC/2 conditions.

To further study the time-killing relationship between gentamicin sulfate and GBP against* Staphylococcus aureus*, the time-killing curves for* Staphylococcus aureus* treated with gentamicin sulfate and GBP-1 (microemulsion, average diameter 90.2 nm) and their mixture under MIC/2 conditions (gentamicin sulfate 2 *μ*g/mL and GBP-1 64 *μ*g/mL and their mixture containing 0.5 *μ*g gentamicin sulfate and 16 *μ*g GBP per mL) are shown in Figure 4. Overall, the bactericidal activity of only gentamicin sulfate was stronger than that of the mixture in the first 6 h. The bactericidal activity of gentamicin sulfate with GBP-1 appeared to be enhanced as the time was increased from 6 h to 24 h. The antibacterial effect of gentamicin sulfate weakened gradually after 12 h was shown in Figure 4. It meant that the concentration of* Staphylococcus aureus* with only gentamicin sulfate was above 10^5^ CFU/mL from 12 h to 24 h. Meanwhile, the antibacterial effect of gentamicin sulfate with GBP-1 could remain a certain antibacterial effect and not to be weak until 24 h (we did not test longer durations). It showed that the concentration of* Staphylococcus aureus* adding gentamicin sulfate with GBP-1 was below 10^4^ CFU/mL from 12 h to 24 h. Moreover, only GBP-1 was always keeping a weak antibacterial effect from the first time to the end of the tested time (24 h). And it showed that the concentration of* Staphylococcus aureus* with only GBP-1 was above 10^8^ CFU/mL in 24 h. We concluded that it enhanced the antibacterial effect and prolonged the antibacterial time of GBP combining with gentamicin sulfate against* Staphylococcus aureus*. Besides, it can be inferred that it could be having the postantibiotic effect (PAE) of gentamicin sulfate with GBP against* Staphylococcus aureus*.

The ability of GBP treatment to sensitize bacterial cells to such a heterogeneous group of antibiotics underlines the nonspecific and potentially general nature of this enhancing activity. We expect that other classes of cytoplasmically targeted antimicrobial agents including food preservatives, biocides, and sanitizers as well as other antibiotics will also show enhanced antimicrobial activities in the presence of GBP. However, because the principal interactions of polyprenols are with the cytoplasmic membrane, it is likely that the effects of membrane-active antimicrobial compounds will be especially enhanced. The results reported here for gentamicin support this theory.

### 3.4. Ca^2+^ Mobilization Assay in* Staphylococcus aureus* Cells Treated with Gentamicin Sulfate and GBP-1 and Their Mixture under MIC/2 Conditions

**Figure 5:**
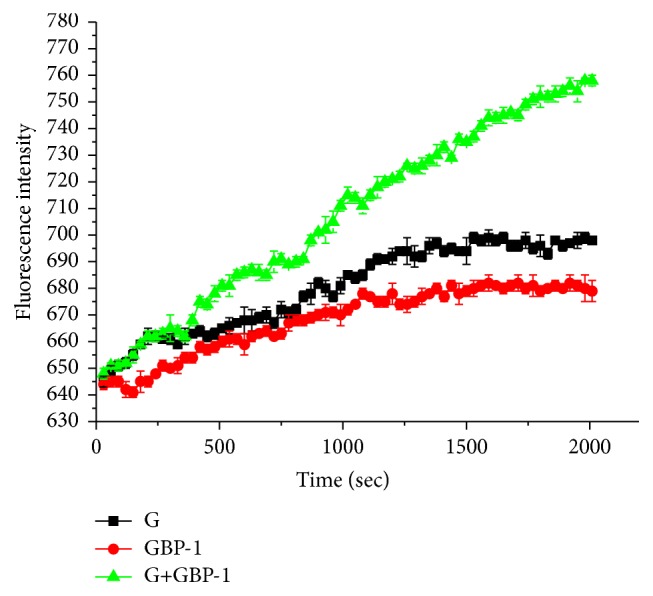
Ca^2+^ mobilization assay in* S. aureus* cells treated with gentamicin sulfate (G) and GBP-1 and their mixture under MIC/2 conditions.

In addition to its interaction with bacterial ribosomes, the cationic aminoglycoside gentamicin has also been shown to have membrane-destabilizing properties [23]. So it is necessary to study the bacteria cell membrane permeability using Ca^2+^ mobilization assay in* Staphylococcus aureus* cells treated with gentamicin sulfate and GBP-1 and their mixture under MIC/2 conditions. From Figure 5, the representative data of Ca^2+^ mobilization assay showed that the mixture group (gentamicin sulfate and GBP-1) exhibited an abrupt increase in fluorescence intensity in contrast to the only gentamicin sulfate or GBP in the tested time. That meant that the mixture group with the bacteria cell showed a rapid increase in intracellular calcium concentrations compared to the only gentamicin sulfate or GBP. From Figure 5, a before-and-after tested comparison showed that a significant increase in intracellular calcium levels was observed in the mixture group (the fluorescence intensity value range was from 648 to 758, *p* < 0.01) compared with the only gentamicin sulfate (the fluorescence intensity value range was from 645 to 699, *p* < 0.05) or GBP (the fluorescence intensity value range was from 644 to 682, *p* < 0.05). It has been confirmed that an increase in intracellular calcium concentrations could be gentamicin-induced cytotoxicity [24]. So there is considerable evidence that GBP microemulsion could enhance the ability of gentamicin inducing an increase in intracellular calcium concentrations to* Staphylococcus aureus*. And that could explain the one reason for synergistic antibacterial activity of GBP microemulsion with the other tested antibiotics.

Gram-negative bacteria tend to be more resistant to antimicrobial agents than Gram-positive bacteria because of the presence of the additional protection afforded by the outer membrane. The outer membrane is more permeable than the cell membrane because of the presence of porin proteins that allow facile permeation of small molecules having below the mass of 500 Da. However, damage to the outer membrane or loosening of its structure by removal of Mg^2+^ results in more facile passage of larger molecules through the cell membrane, resulting in bacterial toxicity [25]. GBP having much longer hydrocarbon tails of the molecules than sesquiterpenoids should be a more effective enhancer of membrane permeability. Besides, unmodified polyprenols are also present in biological membranes of eukaryotic and bacterial [26]. The increased effectiveness of sesquiterpenoids as enhancers of membrane permeability may stem from their structural resemblance to membrane lipids [13]. Krag found nerolidol and farnesol to be more effective as skin penetration enhancers than bisabolol and proposed that the longer hydrocarbon tails of these molecules play an important functional role in promoting greater interaction with the interior of the bilayer than that promoted by other classes of terpenoids [27]. And there is considerable evidence that polyprenols increase membrane fluidity, ion permeability, and propensity of membranes to adopt a nonbilayer, hexagonal II conformation [10]. As a result, it is important to enable preparation of GBP microemulsion which may help some classes of antibiotics to inhibit or kill bacteria.

## Figures and Tables

**Table 1:** Average particle size and zeta potential of the four variants of GBP microemulsion selected.

Samples	Range of the particle size (nm)	Average particle size (nm)	Zeta potential (mv)
GBP-1	12.5~388.7	90.2	−55.2
GBP-2	48.0~865.9	289.7	−49.5
GBP-3	121.8~1565.8	632.9	−45.6
GBP-4	2078.4~25422.7	11012.2	−29.8

**Table 2:** Comparison of the inhibition halos among different samples (Tukey's test at 5% probability).

Samples		Diameter of the inhibition halos (mm) ± SEM, *n* = 3	
		*E. coli*	*S. aureus*
GBP	GBP-1	11.1 ± 0.1^a^	11.5 ± 0.1^a^
	GBP-2	11.1 ± 0.1^a^	11.4 ± 0.1^a^
	GBP-3	11.0 ± 0.1^a^	11.4 ± 0.1^a^
	GBP-4	10.5 ± 0.1^b^	10.7 ± 0.1^b^

1	A^(a)^	13.9 ± 0.1^a^	22.7 ± 0.1^a^
	A+GBP-1	13.4 ± 0.1^b^	26.7 ± 0.1^b(b)^
	A+GBP-2	13.0 ± 0.1^c^	26.0 ± 0.1^c(b)^
	A+GBP-3	13.0 ± 0.1^c^	25.6 ± 0.1^d(b)^
	A+GBP-4	13.1 ± 0.1^c^	22.9 ± 0.1^a^

2	C^(a)^	18.2 ± 0.1^a^	25.6 ± 0.1^a^
	C+GBP-1	22.8 ± 0.1 ^b(b)^	28.8 ± 0.1^b(b)^
	C+GBP-2	22.2 ± 0.1^c(b)^	28.1 ± 0.1^c(b)^
	C+GBP-3	21.5 ± 0.1^d(b)^	27.5 ± 0.1^d(b)^
	C+GBP-4	18.2 ± 0.1^a^	25.7 ± 0.1^a^

3	G^(a)^	26.3 ± 0.1^a^	18.3 ± 0.1^a^
	G+GBP-1	29.6 ± 0.1^b(b)^	25.8 ± 0.1^b(b)^
	G+GBP-2	29.0 ± 0.1^c(b)^	24.9 ± 0.1^c(b)^
	G+GBP-3	28.5 ± 0.1^d(b)^	24.4 ± 0.1^d(b)^
	G+GBP-4	26.2 ± 0.1^a^	18.4 ± 0.1^a^

4	E^(a)^	/	26.8 ± 0.1^a^
	E+GBP-1	11.6 ± 0.1^b(b)^	29.5 ± 0.1^b(b)^
	E+GBP-2	10.9 ± 0.1^c(b)^	28.7 ± 0.1^c(b)^
	E+GBP-3	10.5 ± 0.1^d(b)^	28.2 ± 0.1^d(b)^
	E+GBP-4	9.0 ± 0.1^a^	26.3 ± 0.1^a^

5	P^(a)^	34.5 ± 0.1^a^	/
	P+GBP-1	33.9 ± 0.1^b^	13.5 ± 0.1^b(b)^
	P+GBP-2	34.0 ± 0.1^b^	13.1 ± 0.1^c(b)^
	P+GBP-3	34.1 ± 0.1^b^	12.5 ± 0.1^d(b)^
	P+GBP-4	33.9 ± 0.1^b^	10.0 ± 0.1^a^

^(a)^A: ampicillin, C: ciprofloxacin, G: gentamicin sulfate, E: erythromycin, and P: polymyxin B sulfate.

^(b)^Greater and having statistical difference compared with the corresponding antibiotics group (Tukey's HSD test, *p* < 0.05). The same lowercase letters (a, b, c, and d) in the column of the same group indicate no statistical difference (Tukey's HSD test, *p* > 0.05).

**Table 3:** Minimum inhibitory concentration (MIC) of different samples.

Samples	MIC values (*μ*g/mL)	
	*E. coli*	*S. aureus*
GBP-1	128.0	128.0
A	8.0	2.0
A+GBP-1	144.0^(a)^	65.0^(a)^
C	4.0	2.0
C+GBP-1	66.0^(a)^	65.0^(a)^
G	2.0	4.0
G+GBP-1	65.0^(a)^	33.0^(a)^
E	64.0	2.0
E+GBP-1	96.0^(a)^	65.0^(a)^
P	1.0	64.0
P+GBP-1	130.0^(a)^	64.0^(a)^

^(a)^The mixture groups' total mass concentration.

**Table 4:** Fractional inhibitory concentration (FIC) index used to determine the type of interactions.

Samples	FIC index	
	*E. coli*	*S. aureus*
A+GBP-1	3^(c)^	1^(b)^
C+GBP-1	1^(b)^	1^(b)^
G+GBP-1	1^(b)^	0.5^(a)^
E+GBP-1	1^(b)^	1^(b)^
P+GBP-1	3^(c)^	0.8^(b)^

^(a)^Synergistic effect (0 < FIC index ≤ 0.5). ^(b)^Additive effect (0.5 < FIC index ≤ 1). ^(c)^Indifferent effect (1 < FIC index ≤ 4).
